# Tc-99m Renal Scintigraphy in Complex Congenital Systemic Syndromes Associated with Congenital Anomalies of the Kidney and Urinary Tract: A Retrospective Single-Center Study

**DOI:** 10.1055/s-0046-1820100

**Published:** 2026-04-16

**Authors:** Deepa Singh, Pramit Kumar, Suruchi Jain

**Affiliations:** 1 Department of Nuclear Medicine, All India Institute of Medical Sciences, Bhopal, Madhya Pradesh, India

**Keywords:** anorectal malformations, CAKUT, congenital syndrome, renal agenesis, aplasia, prune belly syndrome, Tc-99m DMSA renal scintigraphy, Tc-99m EC renal scintigraphy, VACTERL association

## Abstract

**Objectives:**

Congenital anomalies of the kidney and urinary tract (CAKUT) result from embryonic developmental defects and are usually not isolated. We evaluate the functional status of the kidney with Tc-99m renal scintigraphy in CAKUT patients associated with a rare complex systemic syndrome/sequence.

**Materials and Methods:**

We retrospectively screened 788 Tc-99m EC (ethylenedicysteine) and 187 Tc-99m DMSA (dimercaptosuccinic acid) renal scintigraphies performed between January 2022 and November 2024. Patients were selected based on the presence of congenital systemic syndrome and CAKUT.

**Statistical Analysis:**

The Kruskal–Wallis test was used to compare grading of functional impairment of kidneys across different systemic syndromes. The Mann–Whitney U-test was used to compare the functional impairment of the kidney for continuous variables.

**Results:**

A total of 38 patients (23 males, 15 females, mean age: 6.7 ± 6.7, range 0.03–33 years) of congenital systemic syndrome with associated CAKUT were identified. The most common systemic syndrome was anorectal malformation in 15 patients (39.5%). A few patients had features of more than one syndrome. The most common CAKUT was an orthotopic or ectopic nonfunctioning kidney in 15 patients (39.5%). The Tc-99m renal scintigraphies showed abnormal findings in 31 patients and normal findings in 7 patients. The proportion of abnormal findings was 81.6% (95% confidence interval: 65.7–92.3%). The difference in proportion of abnormal findings of renal scintigraphy across different syndromes was not statistically significant (Fisher's exact test, *p* = 0.128). The Kruskal–Wallis H-test revealed significant differences in the grading of functional impairment of kidneys across different systemic syndromes, H(16) = 27.24, *p* = 0.039. The Mann–Whitney U-test revealed significant difference ( *p* = 0.007) in serum creatinine of normal Tc-99m scintigraphy (mean = 0.49, *n* = 7) and abnormal Tc-99m scintigraphy (mean =1.25, *n* = 31), U = 36.5, *z* = 2.71, *r* = 1.65. However, there was no significant difference ( *p* = 0.097) in the age of patients who showed normal and abnormal Tc-99m scintigraphies.

**Conclusions:**

A complex systemic syndrome may have occult CAKUT that remains silent initially but presents as a complicated case later. Thus, these patients need functional evaluation with Tc-99m EC and Tc-99m DMSA scintigraphies for early diagnosis of renal anomaly and timely management and follow-up, especially after surgical intervention, to avoid future complications.

## Introduction

The congenital anomalies of the kidney and urinary tract (CAKUT) encompass a broad spectrum of embryonic developmental disorders resulting in kidney and urinary outflow tract defects. CAKUT ranges from mild asymptomatic malformation to severe life-threatening anomalies, with a wide range of prevalence of 4 to 60 per 10,000 live births. 1 2 The congenital renal anomalies may be ectopic positioning, horseshoe, obstructive, or nonobstructive dysplastic or hypoplastic, and nonfunctioning or aplastic kidney. The ureteric anomalies may be duplication, megaureter, pelvic-ureteric junction obstruction (PUJO), vesico-ureteric junction obstruction (VUJO), or ectopic ureteral opening. 3 Urinary-bladder anomalies, such as exstrophy or neurogenic bladder, may cause vesico-ureteral reflux (VUR), and urethral anomalies, like posterior urethral valve (PUV), may lead to ureteral dilation and hydronephrosis (HDN). Ultimately, these lower urinary tract anomalies can lead to renal parenchymal damage and renal failure if not managed in a timely manner. 4 5 The CAKUT may not be an isolated (nonsyndromic) anomaly and found to be associated with rare complex congenital systemic syndromes or sequences, such as anorectal malformation (ARM), VACTERL (the abbreviation for vertebral [V], anorectal [A] and cardiac [C] malformation, tracheoesophageal [TE] fistula/atresia, renal [R] anomalies, and limb [L] malformation), caudal regression, and Mayer–Rokitansky–Küster–Hauser (MRKH) syndrome, etc. 6 7 8 9 The CAKUT may be an asymptomatic component of these complex systemic syndromes in infancy and childhood, and remains unnoticed. However, it may slowly progress and present as a symptomatic or complicated case later.

Technetium-99m (Tc-99m) radionuclide renal scintigraphy is used to assess the functional status of the kidneys, detecting early changes and facilitating timely intervention to prevent chronic renal failure and end-stage renal disease. Also, initial functional imaging serves as a baseline for follow-up of CAKUT patients after any surgical intervention during management of urinary or associated complex syndromes. Tc-99m ethylenedicysteine (EC) is used to evaluate perfusion, parenchymal function, and drainage of the kidney. 10 Tc-99m dimercaptosuccinic acid (DMSA) is actively taken up by the proximal convoluted tubules in the renal cortical parenchyma and remains there for a long time with minimal excretion. Thus, it provides a high target-to-background ratio and can localize even in very poorly/practically nonfunctioning small-sized, dysplastic, malformed, or ectopic kidneys. Additionally, it is helpful to observe the differential function and detect pyelonephritic changes even before the appearance of typical cortical scar. 10

This study aimed to evaluate the functional status of the kidneys using Tc-99m renal scintigraphy (Tc-99m EC and Tc-99m DMSA) in patients with congenital complex systemic syndromes or sequences associated with CAKUT.

## Materials and Methods

### Study Design

This retrospective single-center study was conducted in the Department of Nuclear Medicine at a tertiary care hospital. We evaluated 788 Tc-99m EC (performed for evaluation of renal function and drainage) and 187 Tc-99m DMSA renal scintigraphies (for evaluation of function and pyelonephritic changes/cortical scars) between January 2022 and November 2024. The study was reviewed and approved by the institutional ethics committee. Among 975 Tc-99m renal scintigraphies, the eligible patients were identified based on the presence of complex congenital systemic syndrome with associated CAKUT. We excluded the patients who had no known/suspected congenital systemic syndrome and no CAKUT.

### Data Extraction

The enrolled patients' demographic characteristics and biochemical profiles were extracted from hospital records. We evaluated the detailed patient's history (including antenatal history), examination reports, and available investigations like ultrasonography, intravenous pyelography, micturating cysto-urethrography, computed tomography (CT), and magnetic resonance imaging. The Tc-99m renal scintigraphy imaging data were extracted from the Xeleris workstation of the gamma camera (GE Discovery 670 DR dual-head SPECT/CT system).

### Procedure of Tc-99m EC Renal Dynamic Scintigraphy

For adults, a dosage of 185 MBq (5.0 mCi) of Tc-99m EC was administered, while in pediatric patients, the dose was 44 to 148 MBq (1.2–4.0 mCi), given in proportion to their age and weight (according to Webster's formula).

Imaging was conducted under the gamma camera with the patient lying supine, and the camera detector was positioned over the kidneys (abdominopelvic region). A standard posterior position was used for acquisition, and the anterior position was used only in special circumstance. The patient was injected intravenously with a radiopharmaceutical bolus, and a series of dynamic images was captured in a 64 × 64 matrix. First perfusion images were acquired for 60 seconds, followed by the cortical uptake phase for up to 20 to 22 minutes. The static pre-void, post-void, 2-hour, and 4-hour delayed images were acquired with a pixel matrix of 128 × 128.

### Procedure of Tc-99m DMSA Renal Scintigraphy

For adults, a dose of 133.2 MBq (3.6 mCi) and for pediatric patients, a dose of 30–152 MBq (0.8–4.1 mCi) of Tc-99m DMSA were administered intravenously.

Static images of the abdominopelvic region were obtained 2.5 to 3 hours following radiopharmaceutical administration in anterior, posterior, right, and left oblique views.

Single-photon emission computed tomography (SPECT) or SPECT with low-dose CT was also acquired in some patients for precise localization of cortical defects or abnormal tracer distribution.

### Scintigraphy Interpretation

The raw and processed Tc-99m EC and Tc-99m DMSA scintigraphies were independently interpreted by two nuclear medicine physicians. Images were evaluated for size, shape, position, and differential function of the kidneys. Tc-99m EC scintigraphy was interpreted to evaluate parenchymal function and drainage of kidneys, any obstruction at renal or sub-renal level, and any associated VUR. Tc-99m DMSA scintigraphy was evaluated for cortical radiotracer uptake, presence/absence of pyelonephritic changes, and/or cortical scarring.

### Outcome

We analyzed Tc-99m renal scintigraphies for the presence of functional impairment along with the structural anomalies. The differential function was graded as follows: normal (>45%), mild impairment (<45% to 35%), moderate (<35% to 20%), severe impairment (<20% to 5%), and negligible (<5%). We analyzed the proportion of functional impairment of the kidneys and the grading of severity in different systemic syndromes.

### Statistical Analysis

The data were analyzed using IBM SPSS version. 21. Descriptive statistics were used for baseline characteristics. These values were presented as proportions and percentages for categorical variables and mean ± standard deviation for continuous variables. The confidence interval for the proportion of abnormal Tc-99m renal scintigraphy was calculated using the Clopper–Pearson method. Fisher's exact test was used to compare the functional impairment of CAKUT across different systemic syndromes. The Kruskal–Wallis H-test was used to compare the grading of functional impairment of kidneys on Tc-99m renal scintigraphies across different systemic syndromes. The Mann–Whitney U-test was used to compare the functional impairment of the kidneys for continuous variables, such as serum creatinine and patient age. A *p* -value of <0.05 was considered significant.

## Results

Among 975 Tc-99m renal scintigraphies, we analyzed a total of 38 patients (23 males and 15 females, mean age: 6.7 ± 6.7 years, range: 0.03–33 years) with complex congenital systemic syndrome and associated CAKUT. Out of these 38 patients, 2 (5.3%) were adults, and the remaining 36 (94.7%) were pediatric, including 8 infants and 1 neonate. The mean value of serum creatinine was 1.1 ± 1.0 mg/dL (range: 0.3–4.9 mg/dl). The patient's demographic data (according to age groups) and associated systemic syndromes/sequences are listed in Table 1 .

**Table 1:** Characteristics of patients

Variables	Patients in different age groups			
	Infant (birth to 1 year)	Child (1–12 years)	Adolescent (13–18 years)	Adult (>18 years)
**Age, years (mean ± SD, range)**	0.6 ± 0.3 (0.03–0.92)	6.5 ± 2.8 (2–12)	14	29 ± 5.6 (25–33)
**Sex**				
Male	5	15	2	1
Female	4	10	–	1
**S. creatinine (mg/dL) (mean ± SD, range)**	1.2 ± 1.4 (0.4–4.9)	1.0 ± 0.7 (0.3–2.4)	2.7 ± 2.6 (0.8–4.6)	1.1 ± 0.3 (0.9–1.4)
**Congenital systemic syndromes**				
STRICT-VACTERL with high ARM ( *n* = 5)	2	3	–	–
VACTERL-LIKE with high ARM ( *n* = 2)	–	2	–	–
No VACTERL ( *n* = 3)	–	2	1	–
High ARM ( *n* = 5)	2	3	–	–
Low ARM ( *n* = 1)	–	1	–	–
ARM ( *n* = 1)	–	–	–	1
Caudal regression syndrome ( *n* = 3)	–	3	–	–
Down syndrome ( *n* = 2)	1	1	–	–
Down syndrome plus ARM ( *n* = 1)	–	–	1	–
MRKH syndrome ( *n* = 2)	1	–	–	1
Undescended testis with renal dysplasia ( *n* = 4)	1	3	–	–
Prune belly syndrome ( *n* = 1)	–	1	–	–
Bladder exstrophy-epispadias complex ( *n* = 2)	–	2	–	–
Potter syndrome ( *n* = 1)	1	–	–	–
CAKUT with neurological syndrome ( *n* = 3)	1	2	–	–
CAKUT with bone metabolic disorder ( *n* = 2)	–	2	–	–
**Total (** ***n*** ** = 38)**	9 (23.7%)	25 (65.8%)	2 (5.3%)	2 (5.3%)

Abbreviations: ARM, anorectal malformation; CAKUT, congenital anomaly of kidney and urinary tract; MRKH, Mayer–Rokitansky–Kuster–Hauser syndrome; SD, standard deviation; VACTERL, vertebral, anorectal, cardiac, tracheo-esophageal, renal, limb anomalies.

The most common CAKUT was nonvisualization of one kidney in 15 out of 38 patients (39.5%); 12/15 patients had a single functioning orthotopic kidney, and 3 patients had a single functioning ectopic kidney. There were 4/38 patients (10.5%) who had one practically nonfunctioning kidney (very faintly visualized, small-sized kidney with negligible function). The other kidney in these patients was either normal or associated with some pathology. In addition, 16 out of 38 patients (42.1%) had both orthotopic functioning kidneys. Among them, 12/16 patients had either hydronephrotic, hydroureteronephrotic (HDUN), small-sized kidneys, or pyelonephritic changes/cortical scarring. Only 4/16 patients had both morphologically and functionally normal kidneys, and they underwent Tc-99m DMSA scintigraphy with referral diagnosis of VUR. These were postoperative cases of urinary bladder exstrophy–epispadias complex (EEC) in two patients and high ARM in the remaining two patients. The CAKUT anomalies are summarized in Table 2 .

**Table 2:** Congenital anomaly of kidney and urinary tract (CAKUT)

Types of CAKUT	Number of patients ( *n* = 38)	Percentage (%)
Nonfunctioning kidney	12	31.6
Practically nonfunctioning kidney	4	10.5
Solitary functioning ectopic kidney	3	7.9
1 ectopic and other orthotopic kidney	1	2.6
Both nonvisualized kidneys: hypoplastic/nonfunctioning	1	2.6
Duplex malrotated kidney	1	2.6
Horseshoe kidney	1	2.6
Small-sized kidney	1	2.6
Pyelonephritic changes/cortical scar	5	13.2
HDN/HDUN	4	10.5
Pyelonephritic changes/cortical scar with HDN/HDUN	1	2.6
Vescio-ureteric reflux	4	10.5

Abbreviations: HDN, hydronephrosis; HDUN, hydroureteronephrosis.

Both Tc-99m DMSA and Tc-99m EC scintigraphies were acquired in 7 patients (18.4%), only Tc-99m DMSA scintigraphy was acquired in 23 patients (60.5%), and only Tc-99m EC scintigraphy was acquired in 8 patients (21.1%). Out of 38 patients, 31 had abnormal and 7 had normal Tc-99m renal scintigraphies. The proportion of abnormal findings in Tc-99m renal scintigraphies was 81.6% (95% confidence interval: 65.7–92.3%). The difference in proportion of abnormal findings of renal scintigraphy across different syndromes was not statistically significant (Fisher's exact test, *p* = 0.128). However, the Kruskal–Wallis H-test revealed significant differences in the grading of functional impairment of kidneys across different systemic syndromes, H(16) = 27.24, *p* = 0.039. The findings on Tc-99m renal scintigraphies are summarized in Table 3 .

**Table 3:** Tc-99m renal scintigraphy

Systemic syndrome ( *n* = 38)	Tc-99m renal scintigraphy		Grading of function (Tc-99m renal scintigraphy)					CAKUT
	Normal ( *n* = 7)	Abnormal ( *n* = 31)	Normal ( *n* = 7)	Mildly impaired ( *n* = 3)	Moderately impaired ( *n* = 5)	Severely impaired ( *n* = 6)	Negligible function/NVK ( *n* = 17)	
STRICT-VACTERL with high ARM	0	5	0	0	0	0	5	NFK = 3; practically NFK = 2
VACTERL-LIKE with high ARM	0	2	0	1	0	1	0	Solitary ectopic kidney =1; horseshoe kidney = 1
No-VACTERL	0	3	0	0	0	0	3	NFK = 3 (including one crossed fused ectopic)
High ARM	2	3	2	0	1	1	1	NFK = 1; pyelonephritis/scar = 2, VUR = 2
Low ARM	1	0	1	0	0	0	0	Small-sized kidney = 1
ARM	0	1	0	1	0	0	0	HDN/HDUN = 1
Caudal regression syndrome	0	3	0	0	2	1	0	HDN/HDUN = 2; pyelonephritis/scar = 1
Down syndrome	1	1	1	0	0	1	0	Pyelonephritis/scar = 1; one ectopic kidney = 1
Down syndrome plus ARM	0	1	0	0	0	1	0	HDN/HDUN with pyelonephritis/scar = 1
MRKH syndrome	0	2	0	0	2	0	0	Solitary ectopic kidney = 1; pyelonephritis/scar = 1
Undescended testis with renal dysplasia	0	4	0	0	0	1	3	NFK = 2; duplex = 1; practically NFK = 1
Prune belly syndrome	0	1	0	0	0	0	1	NFK = 1
Bladder exstrophy-epispadias Complex	2	0	2	0	0	0	0	VUR = 2
Potter syndrome	0	1	0	0	0	0	1	Both hypoplastic/NFK = 1
CAKUT with neurological syndrome	1	2	1	1	0	0	1	NFK = 1; solitary ectopic kidney = 1; HDN/HDUN = 1
CAKUT with bone metabolic disorder	0	2	0	0	0	0	2	NFK = 1; practically NFK = 1
**Total**	7 (18.4%)	31 (81.6%)	7 (18.4%)	3 (7.9%)	5 (13.2%)	6 (15.8%)	17 (44.7%)	38 (100%)

Abbreviations: ARM, anorectal malformation; CAKUT, congenital anomaly of kidney and urinary tract; HDN, hydronephrosis; HDUN, hydroureteronephrosis; MRKH, Mayer–Rokitansky–Kuster–Hauser syndrome; NFK, nonfunctioning kidney; VACTERL, vertebral, anorectal, cardiac, tracheo-esophageal, renal, limb anomalies.

The Mann–Whitney U-test revealed a significant difference ( *p* = 0.007) in serum creatinine of normal Tc-99m scintigraphies (mean = 0.49, *n* = 7) and abnormal Tc-99m scintigraphies (mean = 1.25, *n* = 31), U = 36.5, *z* = 2.71, *r* = 1.65. However, the test showed no significant difference ( *p* = 0.097) in age of patients who showed normal Tc-99m (mean = 3.2, *n* = 7) and abnormal Tc-99m scintigraphies (mean = 7.5, *n* = 31), U = 64.5, *z* = 1.66, *r* = 1.01.

## Discussion

The primary population in this study consisted of pediatric patients, as congenital complex systemic syndromes with/without CAKUT are usually diagnosed early in life. However, the study also includes a few adult patients.

Embryologically, the development of the urinary system begins at the third week of intrauterine life with the formation of the nephrogenic cord from the intermediate mesoderm. The nephrogenic cord forms the pronephros, mesonephros, and metanephros. While the pronephros regresses completely, the mesonephros elongates to form the mesonephric/Wolffian duct and protrudes as an outgrowth close to its entrance into the cloaca to form the ureteric bud. The ureteric bud undergoes branching to form the collecting system (ureter, renal pelvis, major and minor calyces, and 1–3 million collecting tubules). The metanephric mesenchyme surrounds the ureteric bud and forms nephrons (comprising glomerulus, Bowman's capsule, proximal and distal convoluted tubules, and loop of Henle). The complete kidney develops by the end of 32 weeks of gestation. Transcription factor WT1, expressed by mesenchyme, regulates glial-derived neurotrophic and hepatocyte growth factors to stimulate growth and branching of the ureteric bud. Similarly, fibroblast growth factor and bone morphogenetic protein 7, secreted by the ureteric bud, stimulate proliferation in metanephric mesenchyme. Molecular signal disturbance between the ureteric bud and the metanephric mesenchyme may cause CAKUT. 11 12 The cloaca divides to form urogenital sinus anteriorly and the primitive anorectal canal posteriorly between the fourth and seventh weeks of gestation, and these two are separated by the urorectal septum. Urogenital sinus forms the urinary bladder, the prostatic and membranous urethra in males. 11 12 13

The most common renal finding in the present study was a solitary functioning kidney, while the other kidney was either congenitally absent (renal aplasia) or had negligible function (dysplasia). The most common systemic syndromes associated with CAKUT were ARM and VACTERL. Also, a few patients had features of more than one syndrome.

**Fig. 1:**
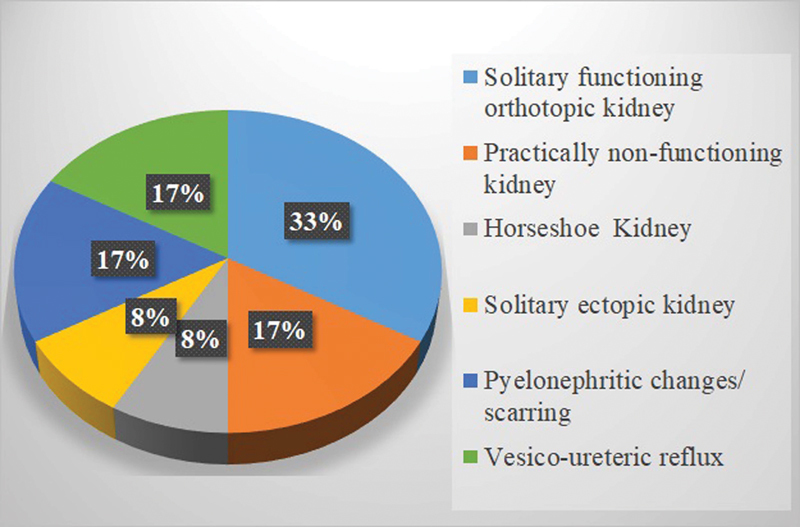
Different congenital anomalies of the kidney and urinary tract in patients with high anorectal malformation ( *n* = 12).

**Fig. 2:**
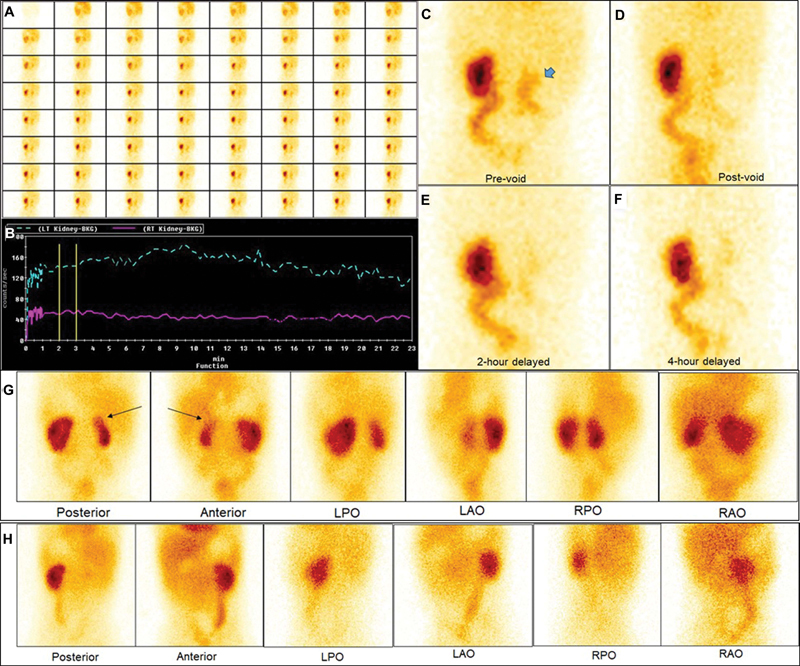
A 10-month-old girl with high ARM syndrome, recto-vestibular fistula, bilateral ectopic ureters, and left megaureter. She underwent anoplasty, colostomy with fistula repair, left ureteric reimplantation, and ureterostomy. The Tc-99m EC scintigraphy ( **a–f** ) shows bilateral hydroureteronephrosis and sluggish drainage, with mildly impaired parenchymal function of the left kidney and severely impaired function of the small-sized right kidney (arrowhead). The initial Tc-99m DMSA scintigraphy ( **g** ) shows pyelonephritic changes/scarring at the upper one-third of the right kidney (arrows). The follow-up Tc-99m DMSA scintigraphy ( **h** ), done at an interval of 1.5 years, shows nonvisualization of the right kidney and no significant change in the left kidney with left gross hydroureter in both initial and follow-up scintigraphies. ARM, anorectal malformation; EC, ethylenedicysteine; DMSA, dimercaptosuccinic acid.

ARM is a rare developmental anomaly involving the distal rectum and anus, and affects 1 to 3 per 5,000 live births. 6 14 According to Krickenbeck's classification, ARM can be low, intermediate, or high type. The low/simple ARM is easily treatable and associated with recto-perineal or recto-vestibular fistula. The intermediate and high ARMs are rare, complex, difficult to manage, and often associated with cloacal anomaly, rectourethral, rectovesical, rectovaginal fistula, or rectal atresia. 15 The intermediate and high ARMs are associated with urogenital, cardiovascular, gastrointestinal, or skeletal system involvement in 30 to 70% patients. CAKUTs are observed in 50 to 60% and 15 to 20% of patients with intermediate/high ARM and low ARM, respectively. 16 Harisankar et al did a study to evaluate the diagnostic role of Tc-99m renal scintigraphy in the management of 40 patients with high ARM and found that the most common renal anomaly was a unilateral nonvisualized kidney. 17 There were 15/38 patients (39.5%) with ARM in the present study. In addition, 12/15 patients had high ARM with various CAKUTs ( Fig. 1 ). One patient with low ARM had one small-sized kidney. Among the remaining two patients with ARM (status nonavailable), one had HDUN and the other had HDUN with pyelonephritic changes/cortical scar. A 10-month-old girl (operated case of high ARM) in the present study showed nonvisualization of the previously seen right kidney with pyelonephritic changes/scarring in the Tc-99m DMSA scintigraphy, signifying severe deterioration in function ( Fig. 2 ).

Apart from the isolated syndrome, few patients with high ARM in the present study were part of the VACTERL syndrome. It can also be associated with minor anomalies. 7 According to EUROCAT classification, VACTERL is of four types: STRICT-VACTERL (three or more major anomalies in different organ systems but without any outside VACTERL association), VACTERL-LIKE (less than three major anomalies and additional minor anomaly to add up to three or more anomalies), VACTERL-PLUS (STRICT-VACTERL or VACTERL-LIKE, with additional anomalies outside of VACTERL association), and NO-VACTERL (less than three anomalies). 18 The kidney and urinary tract involvement is a common finding in VACTERL syndrome. A study done by Cunningham et al in a cohort of 48 patients with VACTERL syndrome found that 33/48 patients (69%) had urinary system involvement, such as VUR, unilateral renal dysplasia, multi-cystic kidney, and duplicated collecting system. They also found that 88% of patients with structural renal manifestations had ARM as an associated feature. 19 In the present study, 5/38 patients with STRICT-VACTERL syndrome with high ARM as a major anomaly had renal dysplasia in the form of a solitary functioning or practically nonfunctioning kidney. Furthermore, 2/38 patients with VACTERL-LIKE syndrome with high ARM had a solitary ectopic kidney with mildly impaired function in one patient, and a horseshoe kidney with severely impaired function in another patient. There were 3/38 patients with NO-VACTERL syndrome, and all of them had a solitary functioning kidney.

Renal ectopia is a condition where a kidney lies outside the renal fossa. Ascent of the kidney from the pelvic region to the abdomen is completed by the ninth week. Simple renal ectopia remains on the same side in the retroperitoneum. Otherwise, it crosses the midline and mostly fuses with the lower end of a normally placed opposite kidney. The incidence of renal ectopia is 1 in 5,000, and rare crossed renal ectopia is 1 in 14,000 pediatric patients. Most cases are incidentally discovered during screening of associated anomalies. Abnormally positioned kidneys may be hypoplastic or dysplastic, with poor outflow and more disease-prone. Ectopic kidneys are associated with abnormalities of the spine in 50% cases, like agenesis of the sacrum, and genital anomalies in 40%, like cryptorchidism, hypospadias, absent vagina, etc. Urinary evaluation is important as VUR may predispose these patients to renal failure. 20 In the present study, Tc-99m renal scintigraphies done in a 6-year-old girl with NO-VACTERL syndrome showed a single large crossed fused ectopic HDUN kidney with VUJO and impaired function, but no scar/pyelonephritic changes. However, follow-up Tc-99m DMSA scintigraphy showed the appearance of pyelonephritic changes.

Apart from ARM and VACTERL, the other syndrome we found in our study was caudal regression syndrome (CRS). CRS occurs due to a defect in the caudal spinal region, with incomplete development of the sacrum in most cases and involvement of the lumbo-thoracic spine in few cases. It is a rare congenital syndrome with an incidence of 1 to 2 per 100,000 live births and occurs more commonly in the babies of diabetic mothers, with 200 to 400 times increased risk. Neonates and infants present with a small-sized pelvis, small and flat buttocks with dimples, and a short intergluteal cleft. 8 21 The incidence of genito-urinary anomalies in CRS is 72%. The most common urinary abnormality is neurogenic bladder; others are renal hypoplasia and dysplasia. 22 A case report by Kesim et al showed no tracer uptake in a bilateral multicystic dysplastic kidney on Tc-99m DMSA scintigraphy in a child with severe CRS. 23 In the present study, a 9-year-old girl with CRS (operated for lumbo-sacral meningomyelocele with VP shunt repair) underwent Tc-99m DMSA scintigraphy for left VUR, which revealed moderately impaired function of the left kidney with multiple cortical scars. A 12-year-old girl with CRS, chronic kidney disease (CKD), and HDUN showed moderately impaired function and sluggish drainage in bilateral kidneys on Tc-99m EC scintigraphy. The other syndromes associated with CRS are VACTERL, MRKH, and OIES (omphalocele, imperforate anus, exstrophy of the bladder, and spinal defect). 9 24

**Fig. 3:**
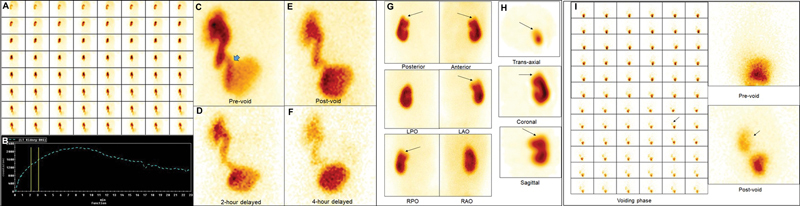
A 3-year-old girl with STRICT-VACTERL and high ARM (blind rectal pouch with recto-vaginal fistula, urethral opening into cloaca), right renal agenesis, caudal regression syndrome (partial sacro-coccygeal agenesis), and MRKH features (bilateral rudimentary Müllerian structure/hemiuterus, single vaginal canal communicating with left-sided Müllerian structure). She underwent colostomy with posterior sagittal anorectoplasty, urogenital mobilization, vaginoplasty, and urethroplasty. The Tc-99m EC ( **a–f** ) and Tc-99m DMSA ( **g** ) scintigraphies showed a single functioning hydroureteronephrotic left kidney with suspicious partial obstruction at the ureterovesical junction (arrowhead in c) and structural anomaly (notching) at the superior pole (arrows in g). The SPECT images ( **h** ) are also consistent with planar DMSA findings. Tc-99m radionuclide Tc-99m DRCG scintigraphy ( **i** ) showed left-sided high-grade vesico-ureteric reflux (arrows). ARM, anorectal malformation; EC, ethylenedicysteine; DMSA, dimercaptosuccinic acid; DRCG, direct radionuclide cystography; SPECT, single photon emission computed tomography.

A 3-year-old girl with STRICT-VACTERL, high ARM, right renal agenesis, CRS, and MRKH features underwent Tc-99m renal scintigraphies. Scintigraphies showed a single functioning HDUN left kidney with suspicious partial VUJO and structural anomaly (notching) at the superior pole. Tc-99m DRCG (direct radionuclide cystography) scintigraphy showed left-sided grade B VUR ( Fig. 3 ).

**Fig. 4:**
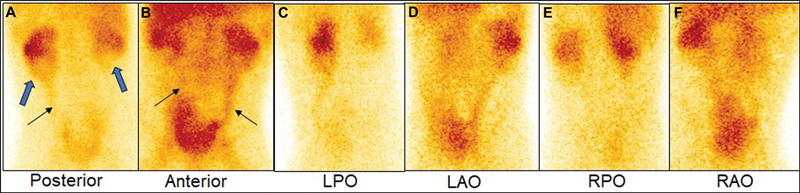
A 14-year-old boy with Down syndrome, ARM (post-operated), and neurogenic bladder presented with bilateral VUR. Tc-99m DMSA scintigraphy ( **a–f** ) showed bilateral gross hydroureteronephrosis (dilated ureter marked with thin arrows) and cortical scarring at the lower poles (thick arrows), with moderately impaired cortical function of the left kidney and severely impaired cortical function of the right kidney. ARM, anorectal malformation; DMSA, dimercaptosuccinic acid.

In the present study, a 14-year-old boy who was a postoperative case of ARM with Down syndrome (DS), neurogenic bladder, and VUR underwent Tc-99m DMSA scintigraphy, which revealed bilateral HDUN, moderate to severely impaired function, and cortical scarring ( Fig. 4 ). DS is the most common chromosomal abnormality, resulting from chromosome 21 trisomy with an incidence of 1 in 920 live births in Indian population. DS patients present with characteristic facial features, intellectual disability with learning impairment, endocrine and immune dysfunction, and congenital cardiac anomalies. 25 Renal involvement in DS is 1.9 to 4.0% patients, which includes hypodysplasia, dilated urinary tract (pyelectasis, megaureter), PUJO, PUV, VUR, and ectopic and horseshoe kidneys. More than half of these anomalies are obstructive. 26 A meta-analysis done by Rossetti et al found that the DS patients have a higher frequency of kidney and urogenital tract abnormalities. 27 Two of our DS patients have raised serum creatinine with CKD. Neonatal comorbidities associated with kidney dysfunction are reduced nephron number, prematurity, low birth weight, and congenital heart disease. 26 28 The long-term complication in DS is CKD from urinary tract infection due to obstructive uropathy, lower urinary tract dysfunction (incontinence, weak stream, frequency), VUR, and neurogenic bladder. 27

In other two patients with DS in the present study, one had an ectopic kidney with congenital heart disease, and the third had a severely impaired, small-sized, contracted kidney with stage 4 CKD. Prevalence of undescended testes is 31 times higher in DS patients than in the general population (6.2 vs. 0.2%, *p* < 0.00001). 27 However, none of our DS patients had undescended testes.

Although urinary and genital systems are two entirely different systems, they develop from a common mesodermal ridge or intermediate mesoderm. The reproductive system development starts at 6 weeks of gestation. The caudal parts of two Müllerian/paramesonephric ducts fuse to form the uterus, cervix, and the upper two-thirds of the vagina, and the cranial parts form the fallopian tubes. The lower one-third of the vagina forms from the urogenital sinus. The Müllerian ducts and urogenital sinus are attached through the sinovaginal bulb. Abnormal expression of several genes (WNT4, RBM8A, TBX6, etc.) is associated with Müllerian duct developmental defect. 29

MRKH is a rare congenital syndrome in females due to Müllerian/paramesonephric duct anomalies, with usually normal ovarian function and karyotype (46, XX). The prevalence of MRKH syndrome is 1 in 5,000 live female births. 30 The type 1 (typical) form is characterized by absence/severe hypoplasia of the uterus, cervix, and upper two-thirds of the vagina. The type 2 (atypical) is more severe and usually has additional abnormalities in the fallopian tubes, ovaries, urological, skeletal, pulmonary, neurological, otological, and cardiovascular systems. 31 32 Type 2 MRKH is also known as MURCS syndrome, which means aplasia/or hypoplasia of Müllerian/paramesonephric ducts (MU), renal dysplasia (R), and cervico-thoracic dysplasia (CS). The most common presentation in these patients is primary amenorrhea. Pelvic pain may occur due to endometriosis in the functioning endometrium of a hypoplastic uterus. The renal abnormalities are reported in 30 to 40% MRKH type 2 syndrome patients, including hypoplastic, aplastic, ectopic, or horseshoe kidney, and may have complications of HDN, acute or chronic pyelonephritis, and renal failure. 9 29 In 40 to 50% of patients with renal agenesis, there is an associated genital abnormality. External genitalia are usually normal with a blind vaginal pouch. There may be an occurrence of renal cell carcinoma in an ectopic or dysplastic kidney. 33 34 35

**Fig. 5:**
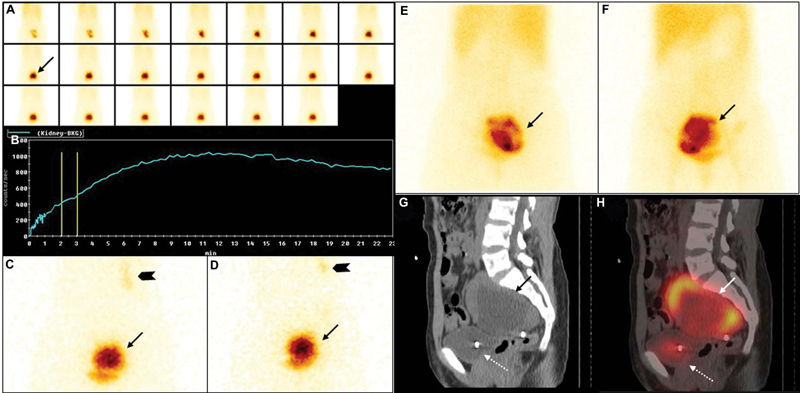
A 25-year-old woman with Mayer–Rokitansky–Kuster–Hauser syndrome. Tc-99m EC dynamic images ( **a** ) and renogram curve ( **b** ) show progressive pooling of tracer in the dilated pelvicalyceal system in a hydronephrotic pelvic kidney with impaired function and minimal drainage (arrow). Delayed static images at 2 hours ( **c** ) and 4 hours ( **d** ) showed significant tracer retention in the dilated pelvis of the kidney (arrow) with faint tracer uptake in the gall bladder (arrowhead). Tc-99m DMSA posterior ( **e** ) and anterior ( **f** ) images showed a single ectopic hydronephrotic pelvic kidney (arrow) with absence of tracer uptake in both the renal fossa. CT ( **g** ) and fused SPECT/CT ( **h** ) sagittal images of the lumbar-pelvic region showed grossly dilated PCS in the pelvic kidney with a DJ stent in situ (arrow) and urinary bladder (broken arrow) with nonvisualization of the uterus. CT, computed tomography; DMSA, dimercaptosuccinic acid; PCS, pelvicalyceal system; SPECT, single photon emission computed tomography.

In the present study, Tc-99m scintigraphies in 25-year-old women with MRKH syndrome showed a single ectopic hydronephrotic pelvic kidney with nonvisualization of the uterus ( Fig. 5 ).

**Fig. 6:**
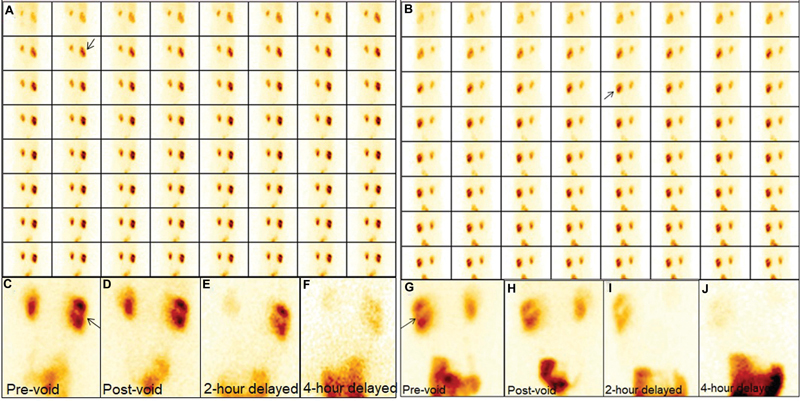
Tc-99m EC scintigraphy in a 9-year-old boy with left undescended testis (post orchidopexy), inguinal hernia, ambiguous genitalia with hypoplastic penis, and posterior urethral valve. The dynamic images ( **a** : posterior, **b** : anterior views) show a small-sized mildly hydronephrotic left kidney with severely impaired parenchymal function. The right kidney is duplex malrotated, mildly hydronephrotic with adequate parenchymal function in both moieties (arrows). The posterior static ( **c–f** ) and anterior static ( **g–j** ) images show sluggish, unobstructed drainage in the small-sized left kidney and both moieties of the right kidney (arrows). EC, ethylenedicysteine.

Similarly, the undescended testis is mostly associated with other congenital anomalies of the genitourinary system. The upper urinary tract anomalies associated with an undescended testis are duplex renal pelvis/proximal ureter, HDN, and multicystic dysplastic, crossed ectopic, horseshoe, and solitary kidneys. These anomalies are usually ipsilateral to the side of the undescended testis. The lower urinary tract anomaly is PUV. In 90% of cases, an undescended testis is associated with an inguinal hernia. 36 37 A 9-year-old boy in the present study with an undescended testis and ambiguous genitalia underwent Tc-99m EC scintigraphy, which revealed a duplex, malrotated, hydronephrotic right kidney with severely impaired function ( Fig. 6 ).

**Fig. 7:**
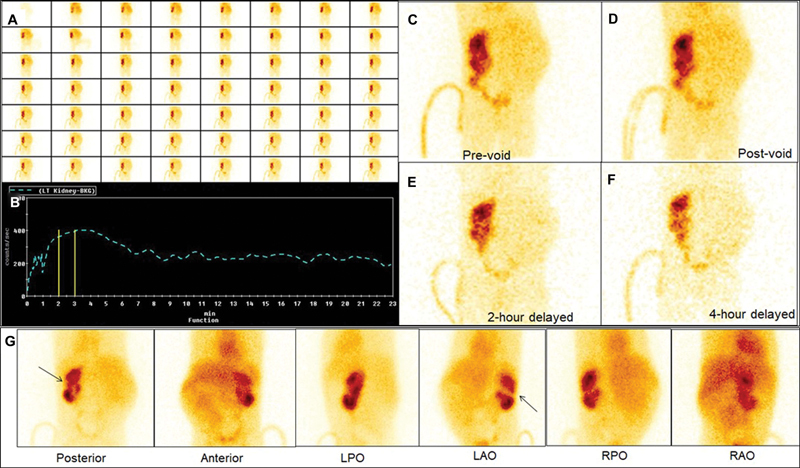
A 5-year-old boy with Prune belly syndrome presented with bilateral undescended testes, thinned anterior abdominal wall muscles, grossly dilated urinary bladder, and hypoplastic right kidney with impaired renal function test (serum creatinine 2.4 mg/dL), deranged hemogram (low hemoglobin), and elevated WBC count possibly due to urinary tract infection. Tc-99m EC dynamic renal scintigraphy ( **a–f** ) shows moderately hydroureteronephrotic left kidney with mildly impaired parenchymal function and slow unobstructed drainage, with nonvisualization of the right kidney. ^99m^ Tc-DMSA scintigraphy ( **g** ) shows no tracer uptake in the right kidney (nonfunctioning kidney) and mildly impaired cortical function with a photopenic defect (cortical scar) in the mid-lateral zone of the left kidney (arrows). DMSA, dimercaptosuccinic acid; EC, ethylenedicysteine.

Out of four of our patients with undescended testes, one also had prune belly syndrome (PBS). PBS is a classical triad of partial or full absence of abdominal wall muscles, abnormalities of the urinary tract, and undescended testis. PBS has an incidence of 1 in 35,000 to 50,000 live births with male predominance (male-to-female ratio of 4.63:1). It occurs due to faulty development of somatic and splanchnic embryonic mesoderm due to altered gene regulation. 38 The most common form of urinary system involvement in PBS is severe nonobstructive urinary tract dilatation. Others are renal dysplasia, calyceal diverticula, scarring, solitary kidney, PUJO, VUR, HDN/HDUN, etc. 39 CKD develops in 8 to 66% patients of PBS, and nearly half of them require renal transplantation. 40 Tc-99m renal scintigraphies in a 5-year-old boy with PBS showed a solitary functioning HDUN left kidney with impaired function, cortical scar, and sluggish drainage ( Fig. 7 ).

EEC also has an abdominal wall defect. It is a congenital malformation that occurs due to abnormal closure of the lower abdominal wall and urinary bladder, with defects in the pelvic bones and floor. It may be associated with genital, renal, spinal, and anal abnormalities. The typical form of EEC is classified according to severity; epispadias is the mildest, and classical bladder exstrophy (CBE) is the most common and severe form, comprising 70 to 80% of the disease. The exstrophy of cloaca is the most severe form and may be associated with the OIES complex. The atypical form of EEC includes duplicated, pseudo, and covered exstrophy. The incidence of CBE is 2 to 4 per 100,000 live births, with male-to-female ratio of 2.4:1. 41 42 Although EEC is a disease involving the urinary bladder, there is 1.9 to 9 times risk of kidney and upper urinary tract involvement. It may be a congenital defect (renal dysplasia) or acquired kidney damage due to infection, obstruction, or during reconstructive surgery. Thus, there is a need for functional radionuclide imaging of the urinary system during initial diagnosis of EEC and follow-up after surgery to quantify functional parenchyma. 43 44 In the present study, there were two postoperative cases of EEC with VUR.

We have one patient of Potter syndrome with CAKUT in the present study, which is a very rare and fatal congenital syndrome with an incidence of 1 in 4,000 fetuses. It was first explained by Edith Potter in 1946. It mainly affects male babies and is associated with premature birth and breech presentation. 45 46 Approximately 95% patients with Potter syndrome/sequence have some congenital renal anomaly like bilateral renal agenesis, cystic renal dysplasia, or obstructive uropathy, leading to reduced amniotic fluid. 47 Compression of the fetus due to oligohydramnios/anhydramnios leads to pulmonary hypoplasia, limb deformities, and typical facial features (Potter facies), characterized by a flattened nose bridge, low-set ears, recessed chin, prominent epicanthal fold, and redundant folds of skin beneath the cheeks. 46 Our patient, a 10-day-old female infant, had nonvisualized kidneys, congenital talipes equinovarus, scoliosis, and flat nose, and Tc-99m DMSA scintigraphy showed absent tracer uptake in the bilateral renal fossa, suggestive of hypoplastic/aplastic kidneys. The patient survived only for a few days following the scintigraphy. Since the outcome of this condition is poor and there are chances of recurrence (3–6%) in subsequent pregnancies, regular antenatal check-ups and interventions for better neonatal outcomes are warranted in mothers with a history of Potter sequence. 45

**Fig. 8:**
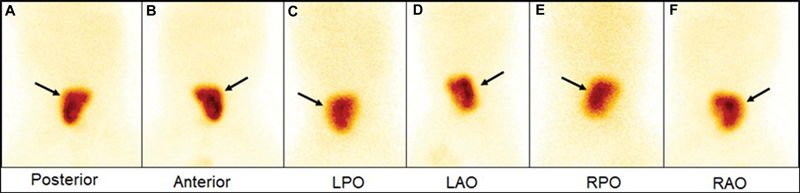
An 8-month-old male child with microcephaly, developmental delay, and subacute malnutrition had vesico-ureteric reflux and mild hydroureteronephrosis. Tc-99m DMSA revealed a single ectopic pelvic kidney (arrow) with adequate cortical function and no scintigraphy evidence of cortical scar/pyelonephritic change ( **a–f** ). DMSA, dimercaptosuccinic acid.

There are a few cases of CAKUT in our study with complex systemic features like microcephaly, developmental delay, bone metabolism disorder, and genital anomalies, etc. An infant with microcephaly and developmental delay showed a single ectopic kidney on Tc-99m DMSA scintigraphy ( Fig. 8 ).

The main limitation of this study was the nonavailability of follow-up data in view of the retrospective study design. Also, there was a limitation of nonavailability of patients' genetic details.

We noticed that there is co-existence of features of different congenital syndromes in the same patient. Also, there were significant differences in the grading of functional renal impairment across different systemic syndromes. These complex systemic syndromes need a multidisciplinary team, as occult CAKUT may present as a complicated case later. The functional evaluation with Tc-99m renal scintigraphy is important for early diagnosis of renal anomaly and follow-up at frequent intervals, especially after any surgical intervention of urinary or associated syndrome, to avoid future complications. Although the descriptive data in the present study provide good insights, a large cohort study with longer follow-up is needed to expand and generalize the results.
